# Hydrochlorate of Cocaine

**Published:** 1885-12

**Authors:** Geo. Watt


### Hydrochlorate of Cocaine.

Read before the Ohio State Dental Society.

BY DR. GEO. WATT.

Debarred from active practice, it is not strange, perhaps, that we have neglected to give this agent a practical investigation, especially as the relaxed and painful state of the nerves inclined us to not only put off till to-morrow that which could be done to-day, but to postpone, indefinitely, all that the force of circumstances failed to drive us into at once. Lately, however, we have given a little attention to the matter, most of our experiments being made with a four per cent. solution.

We got started into the investigation in this way: In the summer a bicuspid tooth became very painful from inflammation in the socket. Dr. Sillito applied the cocaine, and extracted it painlessly. True, it was not hard to extract, but would have been a very severe operation without the medicine.

Further, about a month ago we raised a crop of bastard boils, a cross between boils and abscesses. While passing through the abscess stage of *locomotor ataxie*, we were in the habit of taking nitrous oxide when a very deep abscess was to be cut into. These small affairs seemed not to justify such a course, and yet they were so excessively painful, and on a surface having a high degree of hyperæsthesia, that we lacked the courage to cut into them, "just as they are, without one plea," and so we put about four minims of the four per cent. solution into the hypodermic instrument, placed the point of the needle with the beveled opening lying on the boil, a little to one side of its apex. A gradual introduction of the point between the cuticle and *cutis vera* enabled us to force a minute portion of the solution on the surface of the latter. Waiting a brief period, we then elevated the body of the instrument, and pressed the point of the needle through the skin, at the apex of the tumor. The solution was then forced under the skin, and, in a brief period, a cut through the skin was made, without pain. This was so satisfactory that the entire crop was harvested in the same way. We used from three to five minims of the solution in each operation.

While the preponderance of testimony establishes the local anæsthetic properties of this agent, so many have reported fail ures, that we have tried to find out the cause of such discrepancy. Mistakes in preparation, or the use of inert materials may, in part, explain. But we have seen no notice of careful experiments to determine the time required to produce the anæsthetic state locally, by this drug. One man said he had waited eight minutes, and made partial failure, but suggested that if he had waited twelve, he would have probably succeeded. Others have waited four minutes, and various results are reported. We are inclined to think these delays are too long. At each successive operation, in opening the boils, we reduced the interval between the application and the cutting, and had quite as good, if not better success with the last than with the first.

Then we determined to find out something definite about this. Selecting a highly sensitive surface of sound skin, four minims of the solution were injected immediately under it, and the surface was tested by sudden pricks with the hypodermic needle, deep enough to allow a ready and rapid escape of drops of blood. And this was painless, while similar pricks at a distance were quite painful. At the first trial we waited two minutes, and repeated the experiment day after day, (as we didn't want much of the solution in the blood at once, as it was not known what it might do,) and we kept on till the delay was but thirty seconds.

Next we thought of our Cocker Spaniels. Injecting the solution on the under surface of their ears, we proceeded to interpret their "dog-latin" as faithfully as we could. One of them shrieks from the slightest hurt, except in a fight, and the other is so impatient that he bites, as an argument for release, for equally slight hurts. When introducing the needle, both gave expression to pain, but after twenty seconds with one, and nine seconds with the other, each was quite indifferent to thrusts of the instrument in the vicinity of the point of insertion, even though these thrusts penetrated entirely through the skin. We regard this as valuable testimony, and it seems to indicate that too much time has been spent in waiting for the anæsthetic action of the medicine.

May it not be that in waiting five, and especially eight minutes, the local effects has been, in great part, lost by the absorption of the medicine into the general circulation? Absorption varies in rapidity with the nature of the tissues involved. But, as a general rule, it is more rapid than is usually supposed. Let each reader inject ten to twenty minims of clean water under the skin, where it is reasonably thin, with more than the average quantity of loose cellular tissue beneath it, and watch the process of absorption, and he will receive instruction. The same phenomena will be seen if the injection is made at any point, but tissue, as above described, gives better facilities for observation.

A very intelligent and rather aged physician, who has been a great sufferer from neuralgia in the region of the sacrum, and along the sciatic nerves, writes us that, in his desperation, he had injected ten minims of the four per cent. solution, alternately over the origins of the sciatic nerves, making two injections a day, and that these controlled the pain, and that the relief was noticeable within five minutes after the introduction of the medicine. He had given this treatment but a few trials at the time he wrote to us, but its prompt action, and the uniformity of results thus far, seemed to inspire him with the hope that he would derive some permanent benefit from it.

We hope that carefully conducted experiments will soon tell us more about this valuable medicine. Yea, verily; for we need light.

Since writing the above we repeated an experiment in a modified form. Fifteen minims of water, and five minims of the four per cent. solution were mixed and drawn into the hypodermic instrument, and inserted into the forearm, partly to find out if it would relieve a very severe neuralgia of the occipital nerve, and partly to investigate the local anæsthetic effect. In forty seconds we began thrusting the hypodermic needle into the prominence caused by the injection, passing it quite into the cellular tissue. Eight thrusts of this kind were made within two minutes, and alternately the point of the needle was pressed against a finger-nail, with force enough to have penetrated the skin. The operations were equally painless.

